# Modulating the Substrate Stiffness to Manipulate Differentiation of Resident Liver Stem Cells and to Improve the Differentiation State of Hepatocytes

**DOI:** 10.1155/2016/5481493

**Published:** 2016-01-12

**Authors:** Angela Maria Cozzolino, Valeria Noce, Cecilia Battistelli, Alessandra Marchetti, Germana Grassi, Carla Cicchini, Marco Tripodi, Laura Amicone

**Affiliations:** ^1^Department of Cellular Biotechnologies and Hematology, Section of Molecular Genetics, Sapienza University of Rome, Viale Regina Elena, 324, 00161 Rome, Italy; ^2^National Institute for Infectious Diseases L. Spallanzani, IRCCS, Via Portuense 292, 00149 Rome, Italy

## Abstract

In many cell types, several cellular processes, such as differentiation of stem/precursor cells, maintenance of differentiated phenotype, motility, adhesion, growth, and survival, strictly depend on the stiffness of extracellular matrix that, *in vivo*, characterizes their correspondent organ and tissue. In the liver, the stromal rigidity is essential to obtain the correct organ physiology whereas any alteration causes liver cell dysfunctions. The rigidity of the substrate is an element no longer negligible for the cultivation of several cell types, so that many data so far obtained, where cells have been cultured on plastic, could be revised. Regarding liver cells, standard culture conditions lead to the dedifferentiation of primary hepatocytes, transdifferentiation of stellate cells into myofibroblasts, and loss of fenestration of sinusoidal endothelium. Furthermore, standard cultivation of liver stem/precursor cells impedes an efficient execution of the epithelial/hepatocyte differentiation program, leading to the expansion of a cell population expressing only partially liver functions and products. Overcoming these limitations is mandatory for any approach of liver tissue engineering. Here we propose cell lines as *in vitro* models of liver stem cells and hepatocytes and an innovative culture method that takes into account the substrate stiffness to obtain, respectively, a rapid and efficient differentiation process and the maintenance of the fully differentiated phenotype.

## 1. Introduction

The adult stem cell differentiation choices, as well as the maintenance of the differentiated phenotype, depend on many factors including diffusible molecules and cell/cell and cell/matrix interactions. In recent years an increasing importance has been attributed to the elasticity and the stiffness of the extracellular matrix (ECM), which are physical elements characterizing each adult organ and tissue. These parameters are expressed as resistance to deformation or elastic modulus (*E*) and reported in Pascal units (Pa = newtons/m^2^) [1].

The ECM stiffness is particularly relevant in the liver, the physiology and pathology of this organ being strictly correlated to specific modules of elasticity. In general, it is known that a healthy liver has a low ECM elasticity (0.3–6 kPa), which allows the polarization and the proper functioning of hepatocytes and maintains the quiescence of stellate cells and the optimal fenestration of sinusoidal endothelium. A fibrocirrhotic liver, conversely, is characterized by a significant increase of the matrix elasticity (20 kPa or higher when fibrosis and cirrhosis progress) that, in addition to negatively affecting the microcirculation, can alter the state of differentiation/quiescence of mature liver cells and/or of the stem cell compartment [2]. It has been reported that an increase of liver stiffness may precede the matrix deposition [3], which represents the first response of the organ to several injuries. Therefore, the increase of liver stiffness may also play an important role in the early stages of fibrosis, in addition to characterizing the more advanced disease.

The importance of the substrate elasticity cannot be neglected even in cell cultures. Recently, the impact of the mechanotransduction on many aspects of the behavior of cultured cells has been unveiled. Several cell functions have been proven to be strictly depending on mechanical forces exerted by the extracellular environment, including the execution of the differentiation program, the maintenance of the differentiated phenotype, motility, adhesion, growth, and survival [4, 5]. The effects of an alteration of ECM elasticity on cell behavior are extensive, also affecting the efficiency of DNA uptake [6] (which is particularly important when cells are manipulated for therapeutic approaches or basic research) and the basal transcriptional activity [7].

All these considerations lead to a reassessment of many acquisitions deriving from the studies on cells grown on plastic (with *E* > 1 GPa), since this culture condition would not allow a proper execution of the mechanosensitive biological processes. One of the most common problems of the traditionally performed cell cultures (for all cell types and, in particular, for hepatocytes) is the loss of the fully differentiated phenotype [8] that could be overcome by more physiological culture conditions. Moreover, with respect to stem/precursor cells, the challenge is to find the optimal conditions for their* in vitro* maintenance and expansion, as well as for their quick and correct differentiation [9]. These elements are particularly important in attempting to use cultured liver cells in protocols of cell therapy and liver tissue engineering.

In the recent years, several methods have been developed to culture mammalian cells in a more physiological and efficient setting and important results have been obtained using natural or synthetic substrates with different *E* values.

Gels based on natural ECM components, such as type I collagen, Matrigel, and fibrin, whose stiffness can be modulated by modifying the density of ECM proteins or by chemical crosslinking, allowed impacting tumor growth [10–12] as well as regulating the differentiation and proliferation of normal cells [13].

Recently, to overcome the main limitation of natural ECM (i.e., the limited range of the obtainable stiffness), fully synthetic and covalently cross-linked hydrogels with tunable stiffness have been developed. In a study reported by Pelham Jr. and Wang, polyacrylamide gels of variable stiffness were used for fibroblast cultures [14]. More recently, other synthetic substrates with different *E* values have been utilized [15, 16] to study the effects of mechanical stimuli on the growth and differentiation of several cell types.

Here, we propose the optimization of culture conditions of both liver stem cells and differentiated hepatocytes, using the cellular models deriving from murine livers that we had previously established in line livers and widely characterized both* in vitro* and* in vivo*. The Resident Liver Stem Cells (RLSCs) are immortalized stem cells able to spontaneously acquire features of hepatocytes within several weeks of standard culture [17]. A fully differentiated phenotype was obtained when RLSCs were orthotopically inoculated in growing livers, where they correctly integrated the liver architecture, giving rise to both hepatocytes and mesenchymal liver cells [18]. Concerning the lines of immortalized hepatocytes, they were isolated from livers of both transgenic (MMH/E14) [19] and wild-type (WT/3A) mice [20] and are not tumorigenic, well differentiated, and able to express a wide range of liver functions and products [21–25].

In the attempt to improve the performance of our cells in culture we set up a protocol that, taking into account the stiffness of the substrate, permitted an early and homogeneous differentiation of liver stem cells and, consequently, the analysis, in a restricted time frame, of the molecular events involved. In particular, the use of hydrogels of acrylamide and bisacrylamide with stiffness of 0.4 kPa produced the differentiation of RLSCs into hepatocytes just after 24 hours, whereas higher matrix stiffness (80 kPa) resulted in a substantial maintenance of the fibroblastoid phenotype showing only an initial hepatocyte-specific transcriptional activity. The rapid acquisition of a hepatocyte-like morphology and of a specific gene expression profile on 0.4 kPa hydrogels is correlated with consistent epigenetic modifications on the promoter of the hepatocyte differentiation master gene HNF4*α* and with the lack of activation of molecular pathways, the latter ones being known to respond to mechanic stimuli and involved in cell growth and stemness. Furthermore, the use of a soft hydrogel also allowed hepatocyte cell lines to assume a full epithelial morphology and to express the repertoire of epithelial genes and hepatic functions more effectively, compared to the traditional culture on plastic.

## 2. Materials and Methods

### 2.1. Polyacrylamide Hydrogels

Polyacrylamide hydrogels with two different stiffness values (0.4 kPa and 80 kPa) were prepared on 25 mm glass coverslips (Menzel-Glaser, Thermo Fisher Scientific Inc., MA, USA) using the method described by Li et al. [26] with small changes. In brief, the glass coverslips were treated for 1 hour with 0.2 M HCl and washed four times with water. The same glasses coverslips were treated for 10 minutes with 0.1 M NaOH and washed in water four times. 0.5%_vv_ 3-aminopropyltrimethoxysilane (APTMS 97%; Sigma-Aldrich, St. Louis, MO) was added on glass coverslips for 30 minutes and followed by four water washes. Next, the coverslips were treated with 0.5%_vv_ glutaraldehyde in PBS (glutaraldehyde solution 25%; Sigma-Aldrich, St. Louis, MO) for 1 hour, then washed for 1 hour in water, and air-dried.

In order to obtain hydrogels with 0.4 kPa and 80 kPa, we prepared the following mixture (final concentrations): 3% (for 0.4 kPa) and 16% (for 80 kPa) of Acrylamide (Bio-Rad Laboratories, Inc., Hercules, CA, USA), 0.06% (for 0.4 kPa) and 0.96% (for 80 kPa) of N,N′-methylene bisacrylamide (Sigma-Aldrich), 0.1% APS (ammonium persulfate) (Sigma-Aldrich, St. Louis, MO), and 0.1% of* TEMED* (N,N,N′,N′-tetramethylethylenediamine) (Sigma-Aldrich, St. Louis, MO) in water.

61 *μ*L of this mixture was pipetted on a glass previously treated with hydrophobic silicon polymer (Rain-X) and a coverslip of 25 mm (treated as above) was placed upside down onto the gel droplet. After 30 minutes the coverslip was carefully removed and the polymerized gel in adhesion on it was washed twice with 50 mM HEPES pH 8, cross-linked using 0.05% Sulpho-SANPAH Photoreactive Crosslinker (Thermo Fisher Scientific Inc., MA, USA) in 20 mM HEPES pH 8 under UV_365 nm_ light, and then washed twice with 20 mM HEPES pH 8.

A thin layer of collagen I (GIBCO Life Technology, Monza, Italy) was placed on the hydrogels as follows: 1 mL of collagen 10 *μ*g/mL (in 20 mM acetic acid) was added to the hydrogel in 35 mm plates. After 1 hour of incubation at room temperature the solution was aspirated and the hydrogels were rinsed three times with 1x PBS to remove the acid and cross-linked under UV_365 nm_ light. The hydrogels were then washed once with cold water and blocked with 1% ethanolamine (Sigma-Aldrich, St. Louis, MO) in 50 mM HEPES pH 8 for 30 minutes at 4°C. Finally, gels were sterilized under UV light and incubated with serum-free culture media (Dulbecco's Modified Eagle's Medium and RPMI 1640; GIBCO Life Technology, Monza, Italy).

### 2.2. Cell Cultures

RLSCs [17] were grown in DMEM (Dulbecco's Modified Eagle's Medium) (GIBCO Life Technology, Monza, Italy) with 10% FBS (Sigma Aldrich, St. Louis, MO), 2 mM L-glutamine (Sigma-Aldrich, St. Louis, MO), and antibiotics on collagen I (GIBCO Life Technology, Monza, Italy) coated dishes (BD Falcon, Franklin LAkes, NJ, USA) and on 0.4 kPa and 80 kPa hydrogels obtained as described above. Nontumorigenic murine MMH/E14 and WT/3A hepatocytes [19, 20] were grown in RPMI 1640 with 10% FBS (GIBCO Life Technology, Monza, Italy), 50 ng/mL EGF, 30 ng/mL IGF II (PeproTech Inc., Rocky Hill, NJ, USA), 10 *μ*g/mL insulin (Roche, Mannheim, Germany), and antibiotics, on collagen I (GIBCO Life Technology, Monza, Italy) coated dishes (BD Falcon, Franklin Lakes, NJ, USA) and on 0.4 kPa and 80 kPa hydrogels obtained as described above. 200.000 cells were seeded on each 25 mm coverslip with hydrogel. The morphological analysis was performed by phase-contrast microscopy.

### 2.3. Immunofluorescence

Cells were fixed with 4% paraformaldehyde, permeabilized with 0.2% Triton-X-100 in phosphate-buffered saline, and incubated over night at 4°C with the following antibodies: 1 : 50 goat polyclonal anti-HNF4*α* (C-19 sc-6556, Santa Cruz Biotechnology, USA), 1 : 50 mouse monoclonal anti-E-cadherin (610181, BD Biosciences Pharmingen, USA), 1 : 400 rabbit monoclonal anti-Vimentin (2707-1, Epitomics, USA), and 1 : 50 mouse monoclonal anti-YAP (sc-101199, Santa Cruz Biotechnology, USA). Secondary antibodies are as follows: anti-goat Alexa Fluor 594, anti-mouse Alexa Fluor 488, anti-rabbit Alexa Fluor 488, and anti-mouse Alexa Fluor 594 (all from Molecular Probes, Eugene, OR, USA), diluted to 1 : 500. The nuclei were stained with DAPI (Molecular Probes D1306). Preparations were examined using Nikon Eclipse E600 fluorescent microscope equipped with a 40x objective and a coolSNAP HQ2 CCD camera (Photometrics). Digital images were processed with Adobe Photoshop 7 software (Adobe Systems, Mountain View, CA).

### 2.4. RNA Extraction, Reverse Transcription, and Real-Time Polymerase Chain Reaction (RT-qPCR)

Total RNA was extracted with miRNeasy Mini Kit (Quiagen-GmbH, Hilden, Germany) and reverse-transcribed with iScript cDNA Synthesis Kit (Bio-Rad Laboratories, Inc., Hercules, CA, USA). cDNA was amplified by RT-qPCR using Mini Opticon Real-Time PCR detection system (Bio-Rad) with GoTaq qPCR Master Mix (Promega, Madison, WI, USA). Relative amounts were obtained with 2^−ΔΔCt^ method and normalized to Rpl34.

The list of specific primers is as follows: HNF4*α*: For 5′-TGCGACTCTCTAAAACCCTTGCCG-3′, Rev 5′-GCCCATGGTCAACACCTGCACA-3′; Albumin: For 5′-ACAGACCGGAGGGCTTATCT-3′, Rev 5′-TGGTGTAGACAGGTCAGGATGT-3′; Ttr (Transthyretin): For 5′-GTCCTCTGATGGTCAAAGTC-3′, Rev 5′-CTCCTTCTACAAACTTCTCATCTG-3′; HNF1*α*: For: 5′-AGACCATGTTGATCACAGAC-3′, Rev: 5′-GGGTGGAGATAAAAGTCTCG-3′; Apoc3: For 5′-GGACGCTCCTCACTGTGG-3′, Rev 3′-CACGACTCAATAGCTGGAG-3′; E-cadherin (Cdh1): For 5′-CTACTGTTTCTACGGAGGAG-3′, Rev 5′-CTCAAATCAAAGTCCTGGTC-3′; Snail: For 5′-CCACTGCAACCGTGCTTTT-3′, Rev 5′-CACATCCGAGTGGGTTTGG-3′; Vimentin: For 5′-AGCAGTATGAAAGCGTGGCT-3′, Rev 5′-CTCCAGGGACTCGTTAGTGC-3′; Cyp2b10: For 5′-CAAAGTCCCGTGGCAACTTC-3′, Rev 5′-TCTCCATATTTTTCTCGAAGCTGAA-3′; and Rpl34: For 5′-GGAGCCCCATCCAGACTC-3′, Rev 5′-CGCTGGATATGGCTTTCCTA-3′.

### 2.5. Western Blotting

Cells were lysed in Laemmli buffer 1x (Tris 60 *μ*M pH 6.8, 2% SDS, 10% glycerol, and 5% 2-*β* mercaptoethanol). Equal volumes of extracts were separated by SDS-PAGE and transferred to nitrocellulose membranes (Millipore, MA, USA). Blots were blocked for 1 hour in 5% nonfat milk (Bio-Rad Laboratories, Inc., Hercules, CA, USA) prepared in TBST 1x and incubated overnight with the following primary antibodies: *α*-ERK1 (K23; 1 : 1000) and *α*-CDK4 (c-22; 1 : 1000) (Santa Cruz Biotechnology, Inc., CA, USA), phospho-p44/42 MAPK ERK1/2 (4370; 1 : 1000) (Cell Signalling Technology Boston, USA), and *α*-Vimentin (Clone V9; 1 : 1000) (Millipore, MA, USA). Blots were incubated with HRP-conjugated anti-rabbit secondary antibody (Bio-Rad Laboratories, Inc., Hercules, CA, USA). Immunoreactivity was detected by Enhanced Chemiluminescence Reaction (WESTAR NOVA 2011, Cyanagen, Bologna, Italy) following the manufacturer's instructions.

### 2.6. Cell Cycle Analysis

5 × 10^5^ cells were washed with PBS, fixed in 4 : 1 methanol : acetone, and incubated with RNAse A (2 ng/mL) (Sigma-Aldrich, St. Louis, MO) and with propidium iodide (PI) for 30 min at room temperature. The DNA content was evaluated by flow cytometry with a FACS Calibur (BD Biosciences, USA).

### 2.7. Chromatin Immunoprecipitation (ChIP) Analysis

ChIP analysis was performed as previously reported [27] by using 5 *μ*g of the following antibodies: rabbit anti-H3K4me3 (07473), rabbit anti-H3K27me3 (07449), rabbit anti-H3AcK9 (07352), or rabbit IgG as a negative control (12307) (all from* Millipore*, MA, USA). 5 ng of immunoprecipitated DNA and the relative control were used as templates for RT-qPCR analysis, performed in duplicate. Primers utilized to amplify the HNF1/6 consensus on HNF4*α* promoter are as follows: forward 5′-TCCGAAAGACCCAAGTGTGG-3′ and reverse 5′-GCCAATCACGTCCCAGATCA-3′.

### 2.8. Urea Production/Secretion Analysis

Urea production was analysed in 24 h and 48 h culture supernatants of RLSCs, MMH/E14, and WT/3A cultured on plastic and on 0.4 kPa hydrogels. Urea levels were quantified with a colorimetric urea assay kit (Abnova, CA, USA), according to the manufacturer's instructions. Fresh culture medium was used as negative control.

### 2.9. Statistical Analysis

Data are expressed as the mean ± standard deviation of mean (SD) of three independent experiments (unless stated otherwise) and Student's *t*-test was used for statistical analyses. All the tests were two-tailed and a *p* value < 0.05 was considered statistically significant (*p* value < 0.05 = *∗*, *p* value < 0.01 = *∗∗*, and *p* value < 0.001 = *∗∗∗*).

## 3. Results and Discussion

### 3.1. Soft Substrate Promotes a Rapid and Homogeneous Differentiation of RLSCs toward Hepatocytes

Firstly, we explored the role of substrate stiffness on RLSC self-renewal and differentiation. To this aim, we plated RLSCs on substrate of polyacrylamide gel (hereafter referred to as “gels”) with elastic modulus of 0.4 kPa, matching the intrinsic health liver stiffness, and of 80 kPa, corresponding to the stiffness of fibrocirrhotic parenchyma [2]. As control, RLSCs were grown on the high rigidity substrate of polystyrene dishes (stiffness > 1 GPa). All substrates were coated with type 1 collagen, which has been proven to facilitate adhesion of both stem cells and hepatocytes under the standard culture conditions [17, 19].

**Figure 1:**
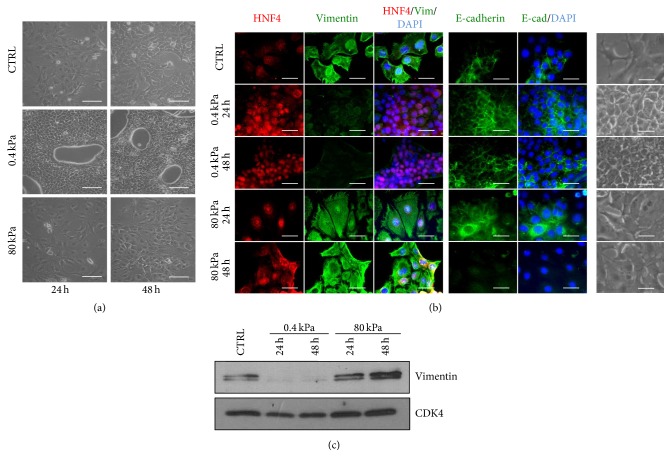
Soft substrate induces a rapid and homogeneous epithelial differentiation of RLSCs. (a) Phase-contrast micrographs of RLSCs grown on Petri plastic dish (CTRL; *E* > 1 GPa) and on hydrogels with *E* = 0.4 kPa and 80 kPa, for 24 and 48 hours. Images are representative of three independent experiments. Scale bar: 100 *μ*m. (b) Phase-contrast micrographs and immunofluorescence of cells cultured on plastic (CTRL), 0.4 kPa and 80 kPa for 24 and 48 hours, stained for HNF4*α*, Vimentin, and E-cadherin. The nuclei were stained with DAPI. Images are representative of three independent experiments. Scale bar: 50 *μ*m. (c) Western blot analysis of Vimentin at 24 and 48 hours after seeding on substrates with the indicated *E* values. CDK4 was used as a loading control.

Since the cell morphology recapitulates the molecular events controlling specific differentiation programs, we first evaluated the morphological changes of RLSCs grown on substrates with different elasticity. The analysis by phase-contrast optical microscopy has revealed that, just after 24 hours of culture on 0.4 kPa gel, cells underwent homogeneous differentiation acquiring a cuboidal shape, establishing tight cell-cell interactions, and being arranged in well-defined epithelial islands surrounding empty spaces (Figure 1(a)). Differently, cells on 80 kPa matrix, as well as cells cultured on plastic, grew interdispersed, maintaining a fibroblastoid shape.

The immunofluorescence analysis confirmed the alternative programs executed by the cells on different stiffness (Figure 1(b)). RLSCs grown on the low-stiffness matrix expressed, just after 24 hours and consistently, the liver-specific transcriptional factor HNF4*α* in the nucleus and the epithelial marker E-cadherin at the membrane (reinforced at 48 hours); at the same time, they lost the expression of the mesenchymal/stem marker Vimentin. Cells cultured on higher stiffness (80 kPa and plastic), instead, did not acquire epithelial/hepatocyte specific markers (only few cells showed a faint HNF4*α* expression) and maintained the expression of Vimentin.

We excluded that the change in Vimentin fluorescence signal was due to the different solubility of the protein when cells grow on surfaces with different stiffness [28], by confirming the immunofluorescence data with a western blot analysis (Figure 1(c)).

**Figure 2:**
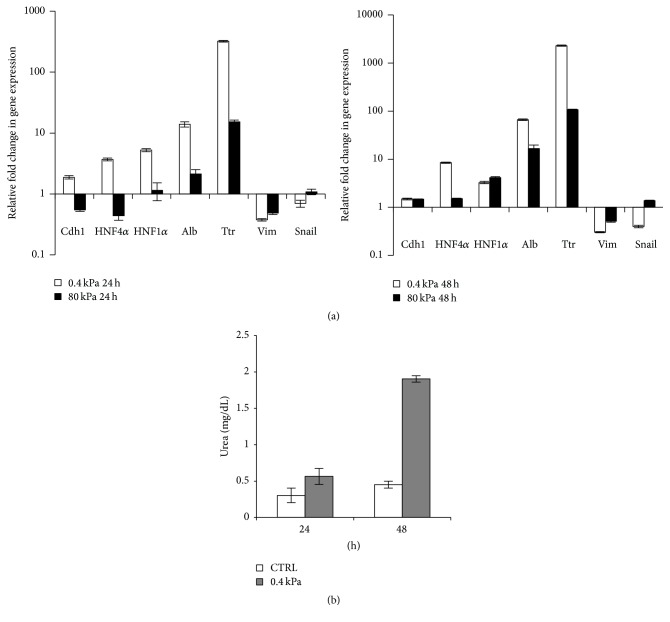
RLSCs grown on soft substrate display epithelial/hepatic gene expression and hepatic function. (a) RT-qPCR analysis for the indicated genes of RLSCs grown on 0.4 kPa and 80 kPa at 24 hours (left panel) and 48 hours (right panel). Data are expressed as fold change in gene expression in cells grown on hydrogels versus CTRL (arbitrary value = 1). The graphics are representative of three independent experiments. Note the logarithmic scale. (b) Urea production in RLSCs. Urea levels in supernatant of cells grown on plastic (CTRL) and on 0.4 kPa hydrogel were analysed at 24 and 48 hours. The mean ± SD of two independent experiments is shown.

A broader analysis of epithelial/hepatocyte and mesenchymal/stemness markers in cells cultured on different stiffness was performed at transcriptional level by RT-qPCR. On a low-stiffness substrate, the epithelial gene E-cadherin (Cdh1) and the hepatocyte-specific genes HNF4*α*, HNF1*α*, Albumin, Ttr, and Cyp2b10 had been strongly expressed just after 24 hours and further upregulated at 48 hours (Figure 2(a)); two additional experiments are shown in Supplementary Figure  1 and the level expression of Cyp2b10 is reported in Supplementary Figure  2A (Supplementary Material available online at http://dx.doi.org/10.1155/2016/5481493). Coherently, the mesenchymal/stem markers Vimentin and Snail were found to be downregulated. On the other hand, the expression of the same genes in cells grown on high-stiffness substrate was only slightly modulated compared to the culture on plastic (Figure 2(a) and Supplementary Figure  1).

Finally, we assessed the differentiation state of RLSCs grown on soft hydrogel evaluating the ability of these cells to synthesize and secrete urea in the culture medium; in fact one of the most important liver functions is the ability to detoxify ammonia transforming this toxic compound into urea.

The levels of urea in supernatants of RLSCs grown on soft hydrogel at 24 and 48 hours were found to be strongly increased compared to the control (RLSCs grown on plastic) (Figure 2(b)).

On the whole, these data reveal that RLSCs already execute an effective program of epithelial/hepatocyte differentiation after 24 hours of culture on a matrix with a low modulus of elasticity and without specific instructive stimuli. Conversely, a hydrogel with high stiffness mostly maintains the RLSC phenotype, delaying the onset of hepatocyte differentiation process.

### 3.2. Soft Substrate Impacts on Signaling Pathways of Mechanotransduction Involved in Hepatocyte Differentiation

The causal link between gel rigidity and differentiation is suggested by early modifications of pathways responsive to extracellular matrix stiffness and involved in stemness/differentiation.

**Figure 3:**
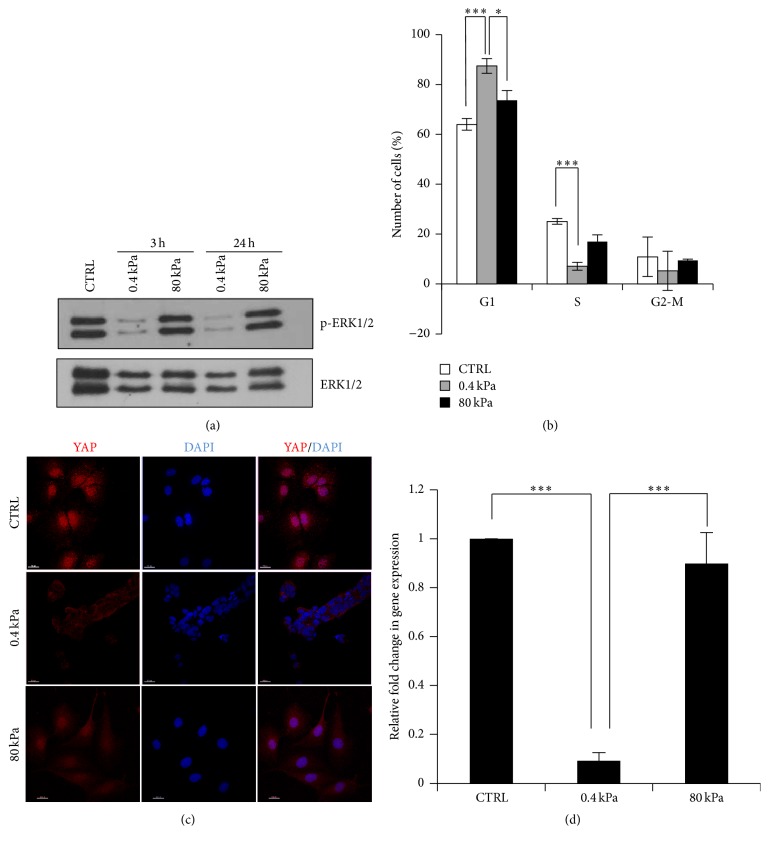
Substrate rigidity controls pathways of mechanotransduction involved in hepatocyte differentiation. (a) Western blot analysis of phospho-ERK1/2 and total ERK1/2 (used as a loading standard) at 3 and 24 hours after seeding on substrates with the indicated *E* values. Images are representative of three independent experiments. (b) Flow cytofluorimetric analysis of cell cycle in RLSCs cultured at the indicated conditions for 24 hours. The values, obtained from three independent experiments, are reported as mean ± SD; ^*∗∗∗*^ *p* ≤ 0.001, ^*∗*^ *p* ≤ 0.05. (c) Immunofluorescence analysis of RLSCs cultured on Petri dish (CTRL), 0.4 kPa and 80 kPa for 12 hours, stained for YAP protein. The nuclei were stained with DAPI. Images are representative of three independent experiments. Scale bar: 20 *μ*m. (d) RT-qPCR analysis of the YAP target gene, Ctgf. Data are expressed as fold change in gene expression in cells grown on 0.4 kPa and 80 kPa for 24 hours versus CTRL (arbitrary value = 1). The mean ± SD of three independent experiments is shown; ^*∗∗∗*^ *p* ≤ 0.001.

The high ECM stiffness is sensed by the focal adhesions/integrin system and consequently activates ERKs, MAP kinases playing a crucial role in regulating many cell functions, including the maintenance of stemness and the dedifferentiation of mature epithelial cells [29–31]. Starting from this evidence, we explored the ERK1/2 activation state in our experimental conditions by means of the survey of its phosphorylated form. As shown in Figure 3(a), the sustained high ERK1/2 phosphorylation levels, observed in RLSCs grown on stiff gel and in standard conditions, appeared to be dramatically reduced in cells cultured on soft gel just after 3 hours and at least until 24 hours of culture.

In accordance with the acquisition of the differentiated phenotype and with the ERK inactivation, the flow cytometry analysis revealed a significant accumulation in the G1 phase of the cell cycle of RLSCs grown on low stiffness, compared to stiff gel or plastic (Figure 3(b)), together with a reduction of the percentage of cells in S phase.

Recently, the identification of the Yorkie-homologues YAP (Yes-associated protein) and TAZ (transcriptional coactivator with PDZ-binding motif) as nuclear relay of mechanical signals exerted by ECM rigidity and cell shape has been reported [32].

Moreover, several data associated these transcriptional factors with the expression of a large number of stemness genes, establishing a critical role of YAP/TAZ in maintaining stem cell pluripotency [33, 34]. Interestingly, recent important reports attributed a crucial role to these molecules also in the liver cell fate: YAP regulates HNF4*α* transcriptional activity during hepatocyte differentiation [35] and its inhibition restores hepatocyte differentiation in advanced HCC [36]. Moreover, an acute inactivation of Hippo pathway signaling* in vivo*, resulting in an elevated YAP activity, has been proven to dedifferentiate adult hepatocytes and to drive liver overgrowth and “oval” cell appearance [37]. Starting from these observations, we analysed the localization of YAP and, accordingly, we observed a nuclear localization of this factor when RLSCs were cultivated on plastic or on high stiffness (where they maintained a fibroblastoid phenotype), while a clear cytoplasmic localization occurred on soft stiffness (where the cells started their epithelial differentiation program) (Figure 3(c)). Consistently with YAP localization, the strong downregulation of its target gene Ctgf [38] was observed in cells cultured on soft gel (Figure 3(d)).

These results correlate the epithelial/hepatocyte differentiation of RLSCs grown on soft substrate with the cytoplasmic localization of YAP and with an inactive ERKs signaling, extending to the liver stem cells what has been observed in other cell systems concerning the role of ECM stiffness in the mechanotransduction and cellular behavior.

### 3.3. Soft Substrate Promotes Early Epigenetic Modifications on HNF4*α* Promoter

Starting from recent reports describing new relationships between biophysical microenvironment, mechanotransduction and epigenetic modifications in cell reprogramming [39–41] and aiming at the identification of new molecular tools for monitoring and/or inducing a rapid and efficient hepatocyte differentiation* in vitro*, we explored the stiffness-induced chromatin modifications on the promoter of HNF4*α* in our cellular model.

**Figure 4:**
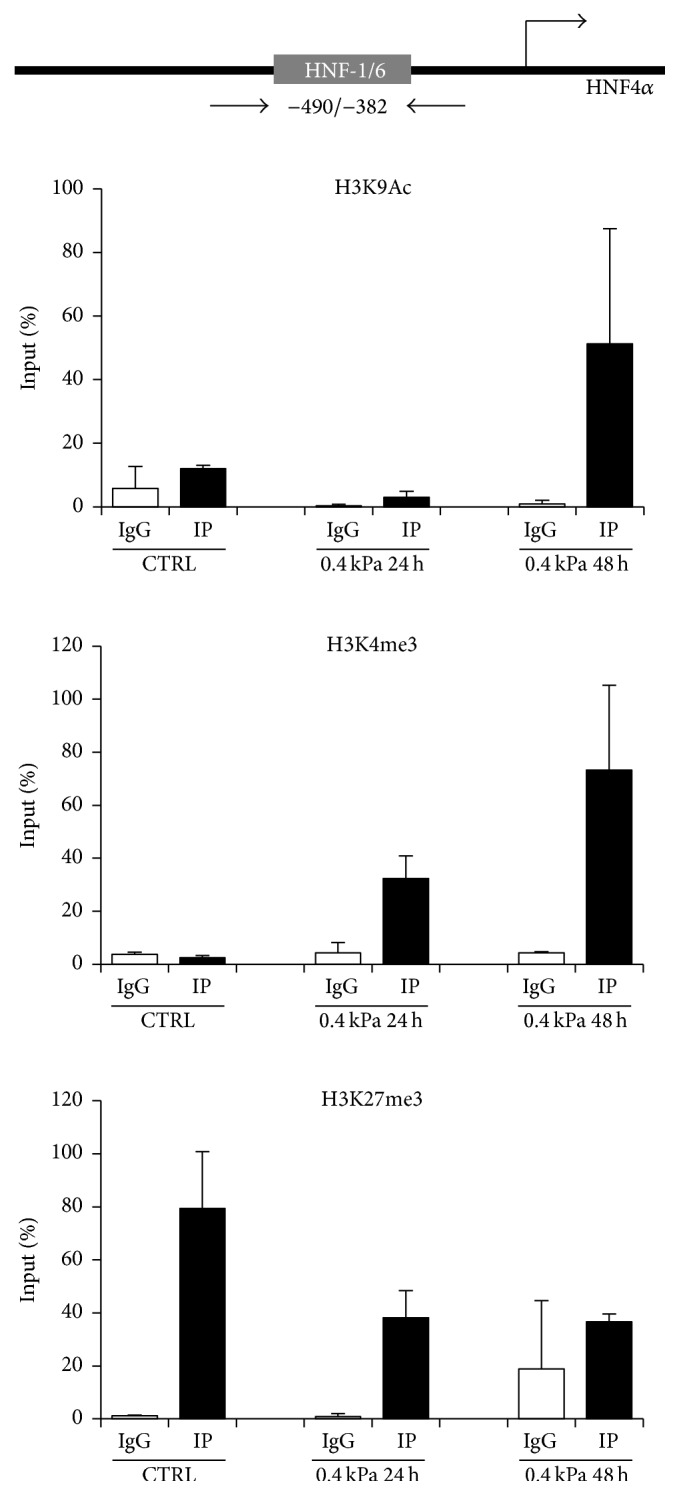
Differentiation of RLSCs towards hepatocytes correlates with early chromatin modifications on HNF4*α* promoter. qPCR analysis of ChIP assay performed to quantify H3K9Ac, H3K4me3, and H3K27me3, on the HNF1/HNF6 binding site of HNF4*α* promoter in RLSCs grown on plastic (CTRL) and on 0.4 kPa hydrogel for 24 and 48 hours. Amplification signals of specific immunoprecipitated samples (IP) and IgG are normalized versus total chromatin (Input) and expressed as % of Input. The mean ± SD of two independent experiments is shown. The upper part of the figure shows a schematic representation of murine HNF4*α* promoter indicating the binding site for HNF1/HNF6 and the relative positions of the qPCR primers.

As shown in Figure 4, the activation of the hepatocyte differentiation program in RLSCs is linked to the early epigenetic modifications of HNF4*α* promoter. In particular, by ChIP assays, we detected an increase in the level of the acetylation of lysine 9 of histone H3 (H3K9Ac, known to be an activating chromatin modification) at the binding site for the transcriptional activators HNF1 and HNF6 [42], at 48 hours from the cell seeding on soft substrate, compared to cells grown on plastic. At the same site, and even earlier (at 24 hours), we have highlighted the increase of histone H3 Lys4 trimethylation (H3K4me3), a histone modification associated with active transcription. Conversely, in the same region, we observed a significant decrease in the level of histone H3 Lys27 trimethylation (H3K27me3), an epigenetic modification known to be correlated with a transcriptional inhibition. This decrease was also observed at 24 hours after seeding on low stiffness, thus preceding the increase of H3K9Ac level.

These results are in accordance with the early transcriptional activation of HNF4*α* observed in soft substrate and confirm that matrix stiffness can impact the epigenetic regulation during stem cell differentiation. Furthermore, the identification of early chromatin modifications involved in differentiation of precursor cells can contribute to the development of new protocols of cell reprogramming and differentiation based on the use of small molecules able to inhibit or activate specific chromatin modifiers, as discussed by Lin and Wu [43].

### 3.4. Soft Substrate Efficiently Sustains the Differentiation State of Cultured Hepatocytes

Obtaining a fully differentiated phenotype of immortalized hepatocytes in culture, together with the maintenance of differentiation of freshly isolated hepatocytes, represents a further challenge for the cellular biologists. It has been largely observed, indeed, that a number of liver-specific functions are progressively lost when hepatocytes are cultivated. These phenotypic modifications are primarily the result of fundamental changes in gene expression concomitant with a decreased transcription of the relevant liver-specific genes and can be interpreted as a “dedifferentiation” of the isolated hepatocytes [8]. To investigate whether soft substrate can sustain or improve the differentiation state of hepatocytes, we utilized hepatocyte cell lines derived both from livers of MMH mice (MMH/E14) [19] and from WT murine livers (WT/3A) [20].

**Figure 5:**
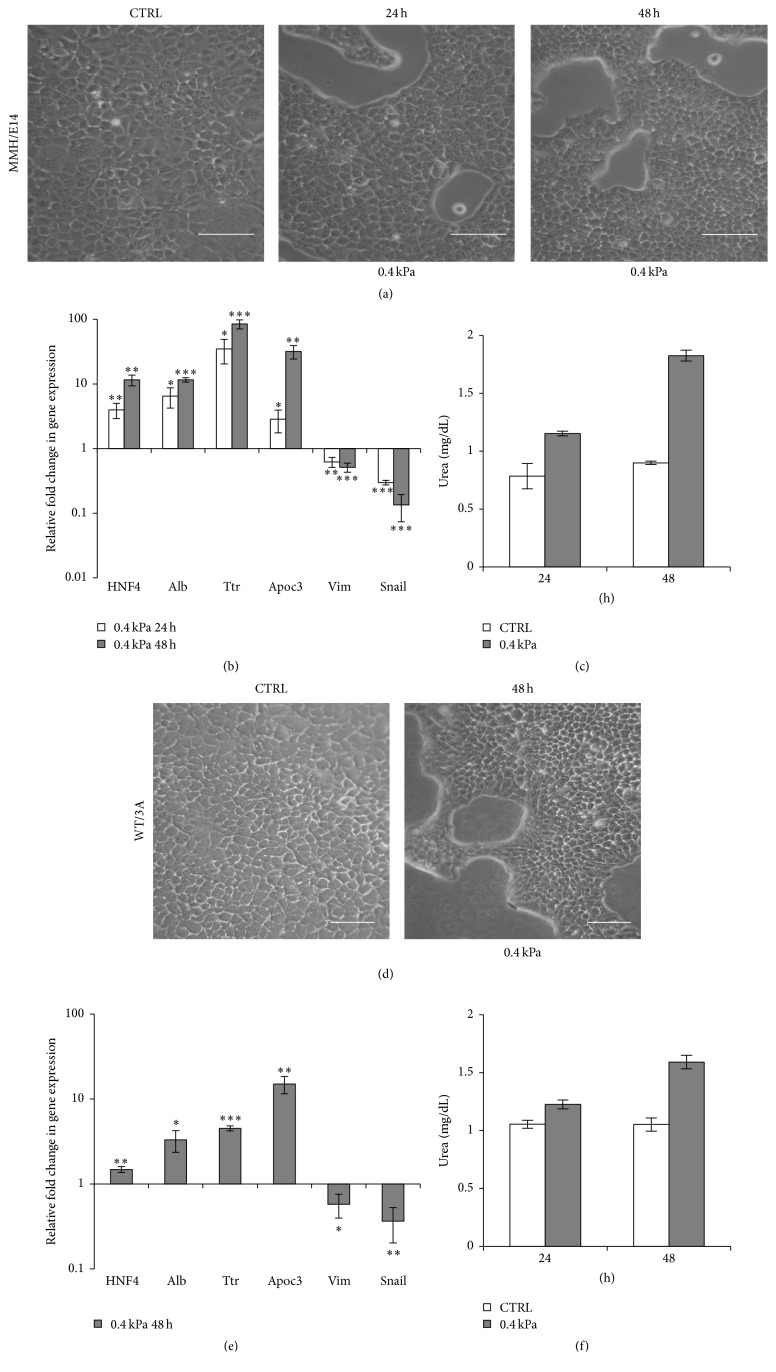
Soft substrate improves the differentiation state of hepatocyte cell lines. (a) Phase-contrast micrographs of MMH/E14 hepatocyte cell lines grown on plastic (CTRL) and on 0.4 kPa hydrogel for 24 and 48 hours. Images are representative of three independent experiments. Scale bar: 100 *μ*m. (b) RT-qPCR analysis for the indicated genes. Data are expressed as fold change in gene expression in cells grown on 0.4 kPa versus CTRL (arbitrary value = 1). The graphic represents the mean of three independent experiments ± SD. Asterisks indicate *p* values in Student's *t*-test (^*∗*^ *p* < 0.05, ^*∗∗*^ *p* < 0.01, and ^*∗∗∗*^ *p* < 0.001). Note the logarithmic scale. (c) Urea production in MMH/E14 hepatocytes. Urea levels in supernatant of cells grown on plastic (CTRL) and on 0.4 kPa hydrogel at 24 and 48 hours were analysed. The mean ± SD of two independent experiments is shown. (d) Phase-contrast micrographs of WT/3A hepatocyte cell lines grown on plastic (CTRL) and on 0.4 kPa hydrogel for 48 hours. Images are representative of three independent experiments. Scale bar: 100 *μ*m. (e) RT-qPCR analysis for the indicated genes. Data are expressed as fold change in gene expression in cells grown on 0.4 kPa versus CTRL (arbitrary value = 1). The graphic represents the mean of three independent experiments. ^*∗*^ *p* < 0.05, ^*∗∗*^ *p* < 0.01, and ^*∗∗∗*^ *p* < 0.001. Note the logarithmic scale. (f) Urea production in WT/3A hepatocytes. Urea levels in supernatant of cells grown on plastic (CTRL) and on 0.4 kPa hydrogel at 48 hours were analysed. The mean ± SD of two independent experiments is shown.

As shown in Figure 5, hepatocytes MMH/E14 (Figure 5(a)) and WT/3A (Figure 5(d)) cultured on 0.4 kPa acquired a more noticeable and homogeneous epithelial phenotype just at 24 hours after seeding: the cells are organized in epithelial islands with the typical cobblestone appearance and delimited sharply bounded empty spaces. Remarkably, hepatocytes showed a significant change in the expression profile of hepatospecific genes that appeared to be strongly upregulated and a significant downregulation of mesenchymal genes that were still expressed in cells cultured on plastic (Figures 5(b) and 5(e); levels of expression of Cyp2b10 are reported in Supplementary Figures  2B  and  2C). The detoxification hepatic function was also analysed evaluating the ability of hepatocytes to synthesize and secrete urea. In both MMH/E14 (Figure 5(c)) and WT/3A (Figure 5(f)) cell lines grown on soft hydrogel, the levels of urea production appeared to be increased compared to the control (hepatocytes grown on plastic).

Thus, while a lot of liver functions are lost when hepatocytes are cultivated in standard conditions, a large repertoire of genes for liver products and hepatic functions (e.g., urea production) can be efficiently expressed and maintained when the cells are cultivated on soft extracellular matrix. These results appeared particularly relevant because they could contribute to the improvement of the reliability of liver cell lines and to the promotion of the culture of differentiated hepatocytes, allowing the maintenance of a functional differentiated state.

## 4. Conclusions

The intent of this work is to contribute to addressing the pressing needs of cell biology to reproduce in culture, as closely as possible, the physiological conditions that,* in vivo*, sustain the biology of each cell type. In particular, we have developed a method for the cultivation of liver cells that takes into account the influence of the rigidity of the substrate. Indeed, it is now clear that the cultivation of cells on substrates whose elasticity is too different from that of the correspondent organ or tissue may alter their gene expression and, consequently, their morphological and molecular phenotype. The use of hydrogels of acrylamide/bisacrylamide with a finely adjustable rigidity has allowed us to study the cells belonging to a soft tissue, such as the liver parenchyma, in a more mechanically physiologic context. Although the polyacrylamide gels may interfere with the adhesion of proteins within the serum or secreted by cells (which might, otherwise, act as adhesive anchors and, consequently, control cell behavior), the comparison between cells grown on the same type of hydrogel but with different stiffness demonstrated the relevance of substrate rigidity in the hepatocyte differentiation process.

Firstly, by manipulating the stiffness of the substrate, we were able to induce a quick and efficient hepatocyte differentiation of stem/precursor cells. This protocol also allowed analysis and identification, in a restricted time frame, of the molecular events involved in the early phase of the differentiation process. The identified molecules could represent useful tools to guide and control the* in vitro* biological processes, such as differentiation and stemness. In fact, although the real involvement of liver resident stem/precursor cells in hepatic regeneration after chronic injury is still debated [44–49], it remains extremely important to handle* in vitro* the differentiation fate of these cells to obtain a source of functional hepatocytes to be used in protocols of cell therapy and tissue engineering.

Moreover, the new culture protocol is useful for inducing/restoring a fully differentiated phenotype of hepatocyte cell lines. This allows giving a new value to the cell lines as models of their counterparts* in vivo*. In fact, given the difficulty in isolating mature hepatic cells from liver explants and maintaining long term cultures of freshly isolated hepatocytes, the improvement of the methods for the cultivation of immortalized cells has become an urgent need. The culture method and the cell lines shown here represent new tools that can be useful to carry out studies on liver physiology and to realize efficient biological modules of bioartificial livers.

Therefore, the rapid amplification of liver cells (obtainable by standard culture) and their following rapid and efficient differentiation (obtainable by shifting cultivation on soft matrix) could represent an innovative method to culture both progenitor and differentiated liver cell lines.

## Supplementary Material

Supplementary Figure 1. (A) and (B) RT–qPCR analysis for the indicated genes of RLSCs grown on 0,4 kPa and 80 kPa at 24 hours (upper panels) and 48 hours (lower panels) in two independent experiments. Data are expressed as fold change in gene expression in cells grown on hydrogels versus CTRL (arbitrary value = 1). Note the logarithmic scale.
Supplementary Figure 2. RT–qPCR analysis of Cyp2b10 in RLSCs (A), MMH/E14 (B) and WT/3A (C) grown on plastic (CTRL) and on 0,4 kPa hydrogel at the indicated times. Data are expressed as fold change in gene expression in cells grown on hydrogels versus CTRL (arbitrary value = 1).
