# Supplementary material for: Modulating the Substrate Stiffness to Manipulate Differentiation of Resident Liver Stem Cells and to Improve the Differentiation State of Hepatocytes
Source: Stem Cells Int. 2016 Jan 12;2016:5481493. doi: 10.1155/2016/5481493 (PMC4737459; doi:10.1155/2016/5481493)
Supplement: Supplementary file 1 — Supplementary Figure 1. (A) and (B) RT–qPCR analysis for the indicated genes of RLSCs grown on 0,4 kPa and 80 kPa at 24 hours (upper panels) and 48 hours (lower panels) in two independent experiments. Data are expressed as fold change in gene expression in cells grown on hydrogels versus CTRL (arbitrary value = 1). Note the logarithmic scale. Supplementary Figure 2. RT–qPCR analysis of Cyp2b10 in RLSCs (A), MMH/E14 (B) and WT/3A (C) grown on plastic (CTRL) and on 0,4 kPa hydrogel at the indicated times. Data are expressed as fold change in gene expression in cells grown on hydrogels versus CTRL (arbitrary value = 1). [file 5481493.f1.pdf]

Supplementary Figure 1.

(A) and (B) RT–qPCR analysis for the indicated genes of RLSCs grown on 0,4 kPa and 80 kPa at 24 hours (upper panels) and 48 hours (lower panels) in two independent experiments.

Data are expressed as fold change in gene expression in cells grown on hydrogels versus CTRL (arbitrary value=1). Note the logarithmic scale.

Supplementary Figure 2.

RT–qPCR analysis of Cyp2b10 in RLSCs (A), MMH/E14 (B) and WT/3A (C) grown on plastic (CTRL) and on 0,4 kPa hydrogel at the indicated times.

Data are expressed as fold change in gene expression in cells grown on hydrogels versus CTRL (arbitrary value=1).

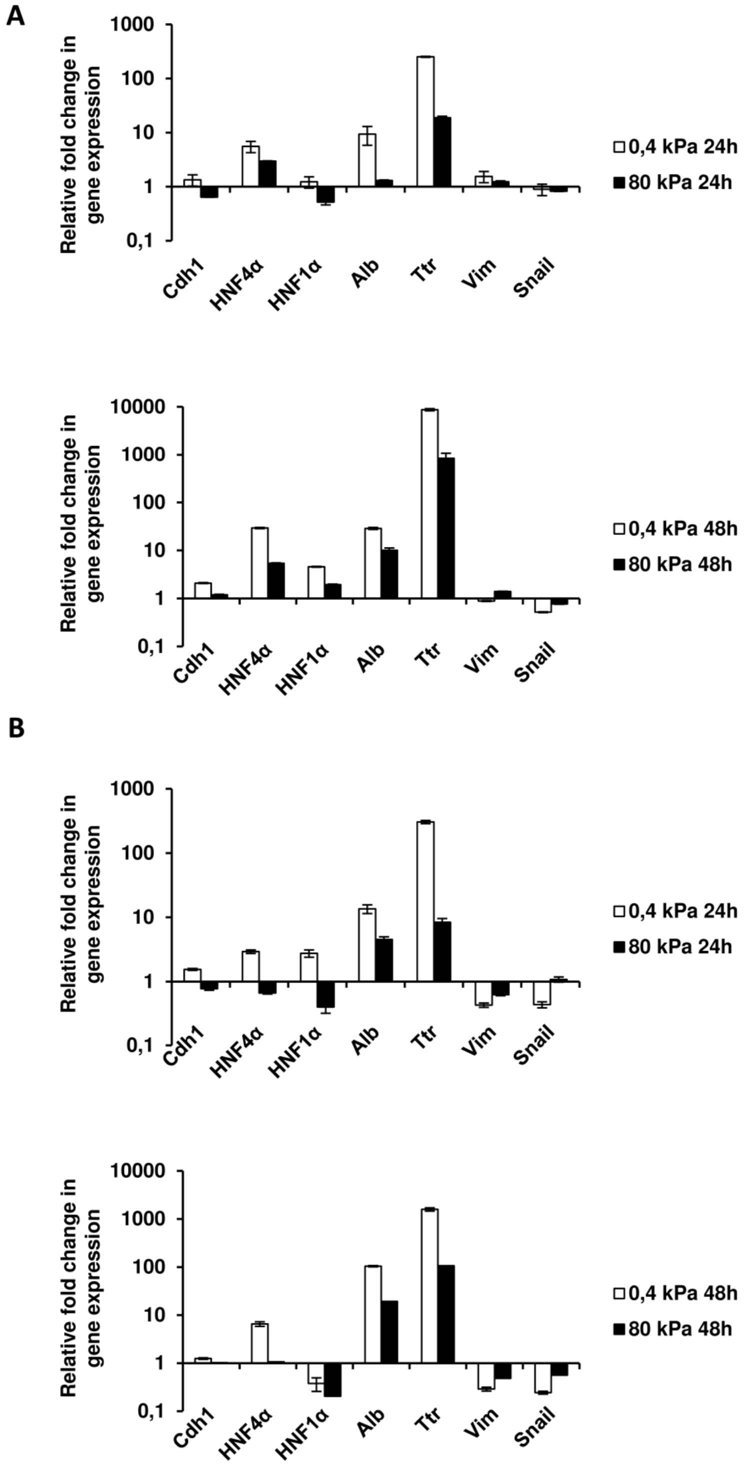

Supplementary Figure 1

A

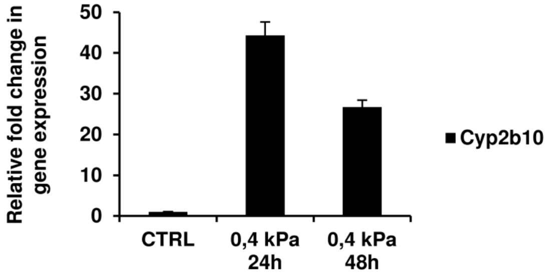

B

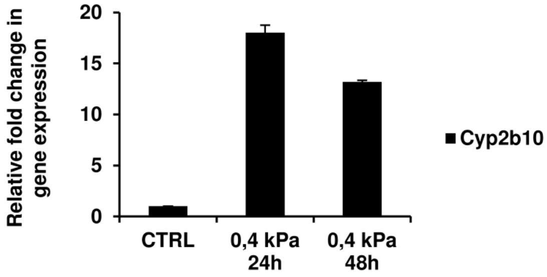

C

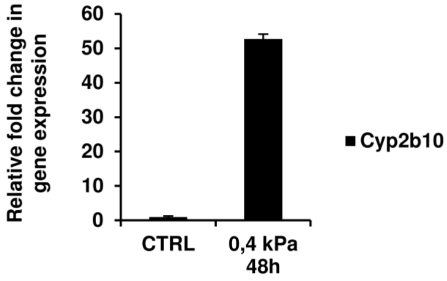

Supplementary Figure 2
